# All 3D Printed Stretchable Piezoelectric Nanogenerator for Self-Powered Sensor Application

**DOI:** 10.3390/s20236748

**Published:** 2020-11-26

**Authors:** Xinran Zhou, Kaushik Parida, Oded Halevi, Shlomo Magdassi, Pooi See Lee

**Affiliations:** 1 School of Materials Science and Engineering, Nanyang Technological University, 50 Nanyang Avenue, Singapore 639798, Singapore; xinran002@e.ntu.edu.sg (X.Z.); kparida@ntu.edu.sg (K.P.); oded.halevi@ntu.edu.sg (O.H.); 2 Singapore-HUJ Alliance for Research and Enterprise (SHARE), Nanomaterials for Energy and Water Nexus (NEW), Campus for Research Excellence and Technological Enterprise (CREATE), 1 Create Way, Singapore 138602, Singapore; magdassi@mail.huji.ac.il; 3 Casali Center of Applied Chemistry Institute of Chemistry, The Hebrew University of Jerusalem, Jerusalem 91904, Israel

**Keywords:** 3D printing, piezoelectric, stretchable sensor, nanogenerator, self-powered sensor

## Abstract

With the rapid development of wearable electronic systems, the need for stretchable nanogenerators becomes increasingly important for autonomous applications such as the Internet-of-Things. Piezoelectric nanogenerators are of interest for their ability to harvest mechanical energy from the environment with its inherent polarization arising from crystal structures or molecular arrangements of the piezoelectric materials. In this work, 3D printing is used to fabricate a stretchable piezoelectric nanogenerator which can serve as a self-powered sensor based on synthesized oxide–polymer composites.

## 1. Introduction

Stretchable electronics are becoming essential for the next-generation wearable electronic systems due to their deformability. These can be widely applied in health monitoring, sensing, sports, and many life-quality or well-being improvement purposes. The largest challenge in stretchable electronics is the power supply for portable devices [1]. To realize autonomous electronics such as self-powered sensors, stretchable energy harvesters are required [2,3,4]. A piezoelectric nanogenerator is a kind of energy harvester that converts mechanical energy into electricity by the inherent polarization in the piezoelectric materials. The output power is stable and it can also serve as a self-powered sensor due to the linear relationship of the external force and the output signal [5]. While a 3D printable stretchable triboelectric nanogenerator has been demonstrated [6], there was no report on the fully 3D printable stretchable piezoelectric nanogenerator, due to the lack of printable stretchable piezoelectric ink, challenges in poling the piezoelectric elastomer system, and the lack of stretchable conductive ink for 3D printing.

Nanostructured oxides including nanowires, nanoparticles, and nanoclusters have attracted attention as composite fillers in many research areas such as energy storage, catalysis, sensing, and energy harvesting due to their functional and mechanical reinforcement to the matrix. Barium titanate (BaTiO_3_) is one of the most popular piezoelectric oxides with high piezoelectricity, high dielectric constant, and ferroelectricity. The potential applications of BaTiO_3_ nanocomposites include high energy capacitors which our group published actively [7], sensors [8], bone scaffolds [9], and piezoelectric devices [10,11,12]. In this work, BaTiO_3_ nanoparticles were combined with oligomers of 3D printable elastomers to form a 3D printable piezoelectric ink. Stretchable electrodes were also 3D printed onto the stretchable piezoelectric nanocomposite to fabricate an all 3D printed stretchable piezoelectric nanogenerator.

## 2. Materials and Methods

**Figure 1:**
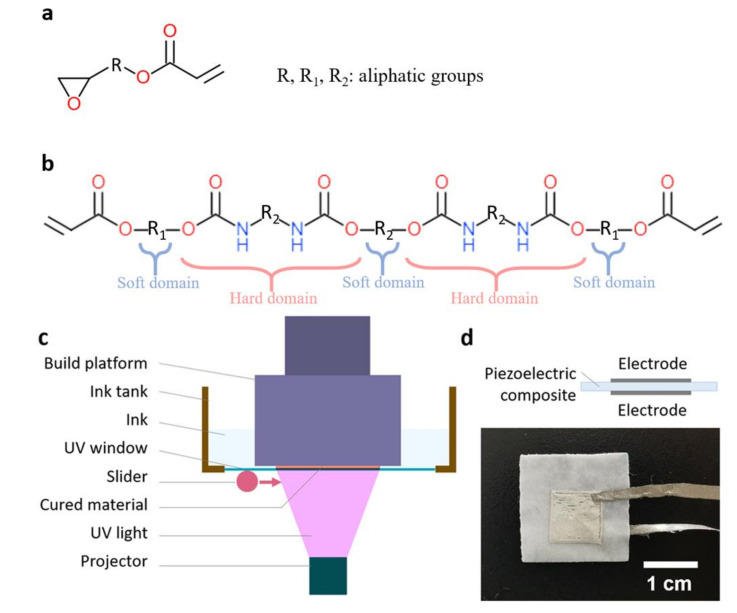
Chemical structure of (**a**) epoxy aliphatic acrylate (EAA); (**b**) aliphatic urethane di-acrylate (AUD); (**c**) schematic of digital light processing (DLP) setup; and (**d**) schematic and photograph of a printed device.

To prepare the stretchable piezoelectric composite, 15 wt % barium titanate nanoparticles (BaTiO_3_ NPs), which were obtained from Nanostructured & Amorphous Materials, Inc., with piezoelectric tetragonal phase and with an average 200 nm diameter, were mixed with photocurable oligomers: 41.65 wt % epoxy aliphatic acrylate (EAA, Ebecryl 8413, Allnex, Frankfurt, Germany) and 41.65 wt % aliphatic urethane diacrylate (AUD, Ebecryl 113, Allnex), and 1.7 wt % photo-initiator diphenyl(2,4,6-trimethylbenzoyl)phosphine oxide (TPO, Sigma-Aldrich, 97%, St. Louis, MO, USA) to form the 3D printable ink [13]. The chemical structures of the EAA and AUD oligomers are shown in Figure 1a,b. The EAA and AUD ink can form a highly elastic elastomer after being cured by UV light with the addition of TPO. Since this ink is photocurable, it was 3D printed with the digital light processing (DLP) system (Asiga, PICO2, Alexandria, Australia) to form a piezoelectric film (Figure 1c). The electrodes were fabricated by a direct-write 3D printer (Hyrel, System 30M, Atlanta, GA, USA) with ethylene–vinyl acetate (EVA) (DuPont, Elvax 40W, Wilmington, NC, USA), chlorobenzene (Sigma-Aldrich, anhydrous, 99.8%), silver flakes (Sigma-Aldrich, 10 µm, ≥99% trace metals basis). The whole device was a sandwich structure with a 1 cm^2^ effective area, as shown in Figure 1d. The crystallographic phase of the materials was characterized by a powder X-ray diffractometer (XRD, Shimadzu XRD 7000, Kyoto, Japan) and the polarization–electric field loop (P–E loop) was performed with Radiant Ferroelectric Premier Equipment, containing Radiant Precision High Voltage Interface and Precision Premier II. The morphology of the materials was characterized by a field-emission scanning electron microscope (FESEM, JEOL 7600F, Akishima, Japan). The mechanical properties were tested by a mechanical tester (Instron 5567, Norwood, MA, USA). The longitudinal mode piezoelectric coefficient (d_33_) of the samples was tested by a standard d_33_ m (Sinocera YE2730, State College, PA, USA), and the energy harvesting performances were measured by a customized shaker system including a function generator (Sinocera YE 1311), a signal amplifier (Sinocera YE5878), a magnetic shaker (Sinocera JZK-20), an oscilloscope (Tektronix MDO 3024, Beaverton, OR, USA), and a low noise current preamplifier (Stanford Research System, Model SR570, Sunnyvale, CA, USA). For the measurement of the energy-harvesting performances, the samples were fixed on the shaker, 60 N force was exerted onto the sample and the output signals were obtained from the oscilloscope. For testing as gait sensing, the packaged device is mounted on human heel under motion for sensing data acquisition. This study was approved by the Institutional Review Board (IRB) at Nanyang Technological University (IRB-2017-08-038).

## 3. Results and Discussion

**Figure 2:**
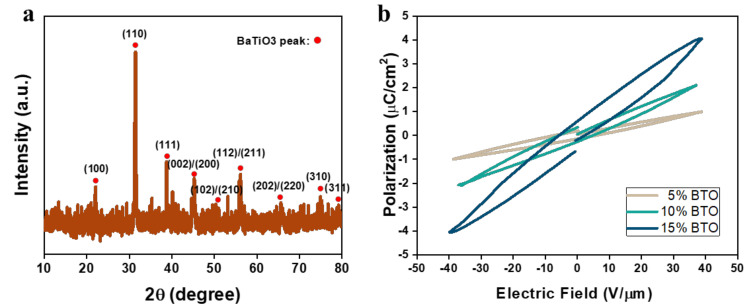
(**a**) XRD pattern of the printed BaTiO_3_ NP/EAA/AUD film; and (**b**) polarization hysteresis loop (P−E loop) of BaTiO_3_ NP/EAA/AUD samples with 5%, 10%, 15% BaTiO_3_ nanoparticles (NPs) by weight.

XRD testing was done on the 3D printed 15 wt % BaTiO_3_ NP/EAA/AUD film (Figure 2a). The film thickness is about 100 µm. The peak at 2θ = 45° splits into two peaks, indicating that the BaTiO_3_ is in the piezoelectric tetragonal phase [14], all other peaks can be assigned to the BaTiO_3_ tetragonal phase in accordance with the International Centre for Diffraction Data (ICDD) PDF #01-074-1965. This indicates that the ink formation and printing process do not affect the crystal structure of the BaTiO_3_ NPs after the UV curing during the 3D printing. 

The ferroelectric hysteresis loop was measured for the BaTiO_3_ NP/EAA/AUD samples with a BaTiO_3_ NP concentration from 5 to 15 wt % as shown in Figure 2b. The saturation polarization and the remanent polarization both increased with the increasing concentration of the BaTiO_3_ NP under the same electric field, indicating the improved piezoelectricity at a higher content of piezoelectric oxides. As the BaTiO_3_ NPs could block the UV light, the ink with more than 15 wt % NPs could not be successfully cured during 3D printing with the same photoinitiator content and the same power of the UV light in the current DLP printer. Thus, 15 wt % BaTiO_3_ NP was chosen to be the optimum concentration for the printable ink in piezoelectric devices. The piezoelectric coefficient of the printed piezoelectric films with 15 wt % BaTiO_3_ NP that is poled under 25 V/μm electric field is measured to be 0.78 pC/N. This value is smaller than the other reported bulk or microscale BaTiO_3_ materials [15,16,17]. This may be due to the damping effect of the 3D printed elastomer matrix, which reduces the force transferred onto the nanoparticle in the d_33_ measurement process. Another possible reason is that the nanoscale BaTiO_3_ particles tend to recover from the aligned dipole moment after poling due to the lack of domain wall restrictions from neighbouring grains [18,19].

**Figure 3:**
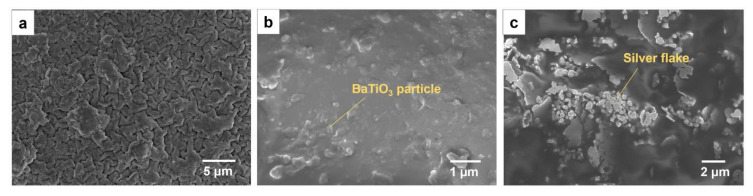
SEM image of (**a**) the BaTiO_3_ NP/EAA/AUD composite surface; (**b**) the cross-section; and (**c**) the Ag flake/ethylene–vinyl acetate (EVA) electrode.

To examine the morphology, SEM imaging was conducted on the sample and the electrode. Figure 3a is the surface of the printed BaTiO_3_ NP/EAA/AUD composite. There are obvious buckling structures throughout the sample surface, which contributed to the large elasticity that was verified later on. During mixing, the nanoparticles with oligomers and air bubbles are easily formed and difficult to remove, which makes the piezoelectric material susceptible to discharge during poling. With the DLP printing system, this problem can be solved using the slider to distribute a thin layer of ink between the window and the substrate and remove the air bubbles from each printing layer as shown in the schematic (Figure S1 in the Supplementary Materials) and the pore-free cross-section image (Figure 3b). 

**Figure 4:**
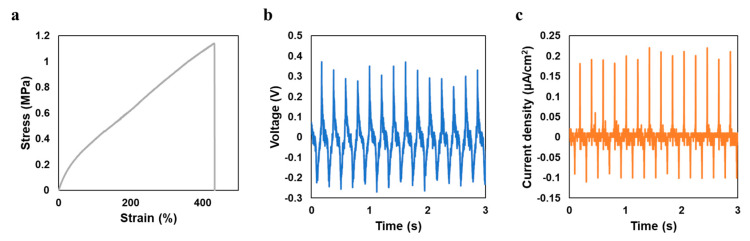
(**a**) Stress–strain curve of the printed BaTiO_3_ NP/EAA/AUD sample with 15 wt % BaTiO_3_ NP content and 2 wt % photoinitiator diphenyl(2,4,6-trimethylbenzoyl)phosphine oxide (TPO), the Young’s modulus is 0.83 MPa up to 434% max strain. This stress–strain curve is obtained from the median of 5 testing results based on maximum tensile strain, with a standard deviation of 27.07%; (**b**) voltage output; and (**c**) the current output of the printed BaTiO_3_ NP/EAA/AUD sample under the compression force of 60 N, 5 Hz.

**Figure 5:**
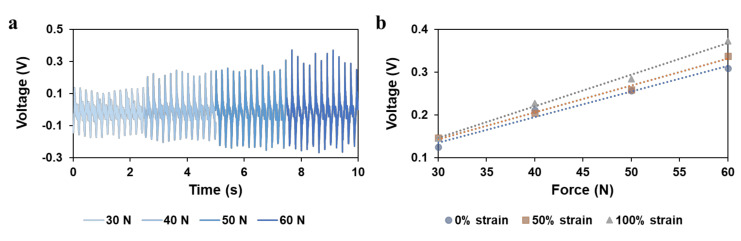
Characterization of sensitivity: (**a**) output voltages under different impact forces ranging from 30 to 60 N; (**b**) average peak output voltage (abstract value) under different impact forces ranging from 30 to 60 N and under different stretching conditions from 0% to 100% strain.

To fabricate a stretchable nanogenerator, stretchable electrodes are required. A highly stretchable and printable conductive ink formulation was reported by Wang et al. based on ethylene–vinyl acetate (EVA), silver flakes, and eutectic gallium indium (liquid metal) particles [20]. This ink system provides stable electric conductivity under as train as high as 1000% and excellent cycling stability. The formulation with 50 wt % silver flakes and 50% EVA solution was adapted for the stretchable electrode in this work. The size of silver flakes ranges from 1 to 5 micrometers. Liquid metal was not added because the liquid metal NPs tend to aggregate under repeated compression, which may cause short circuits. Without the addition of liquid metal, the conductor is also highly conductive (R/R_0_ = 8) under a 200% strain with conductivity before stretching 11,240 S cm^−1^ [20]. The morphology of the stretchable conductor is shown in Figure 3c. Silver flakes are distributed inside the EVA matrix, forming connections among each other to conduct the electricity. The interface between the electrode and the piezoelectric composite is shown in Figure S2 of the Supplementary Materials, indicating that the two materials are in good contact and without interdiffusion. Tensile testing was done on the printed BaTiO_3_ NP/EAA/AUD composite sample with 15% BaTiO_3_ NP content as shown in Figure 4a. The maximum tensile strain for the printed film sample is 434%, and Young’s modulus is 0.83 MPa, in which both the elongation and Young’s modulus increase compared with the previously reported 3D printed EAA/AUD samples without BaTiO_3_ nanoparticles [13], due to the reinforcement of the nanoparticle filler. The maximum strain is higher than all the stretchable piezoelectric materials reported to date [2,3,21,22,23,24,25,26,27,28,29,30]. This higher elasticity could be mainly attributed to the EAA/AUD matrix. According to previous research, the high stretchability of the polymer matrix is due to the presence of hydrogen bonds between hard domains of the AUD. Upon mechanical loading, the breakage of hydrogen bonds dissipates energy and therefore results in the high stretchability of the elastomer system [13]. A cyclic tensile testing was done on the BaTiO_3_ NP/EAA/AUD as shown in Figure S3 of the Supplementary Materials, which further proves the repeatable elastic behaviour of the composite. The energy harvesting behaviour is evaluated by pressing on the printed stretchable BaTiO_3_ NP/EAA/AUD sample with a 1 cm^2^ effective area. As shown in Figure 4b,c, the voltage output under 60 N force under 5 Hz frequency is 0.29 V, and the current density is around 0.20 μA/cm^2^. The power density can be calculated by Ohm’s law, which is 57 nW/cm^2^. The values are comparable to other piezoelectric nanogenerators made of lead zirconate titanate (PZT), zinc oxide (ZnO), and polyvinylidene difluoride (PVDF)-based piezoelectric materials [31,32,33,34,35,36,37], and this power density can be used for powering various types of low energy consumption sensors such as temperature sensors, electrochemical sensors, biosignals sensors, and other mechanical sensors [38,39,40,41,42]. Under 100% strain stretching, the voltage and current output slightly increased to around 0.38 V and 0.23 μA/cm^2^ at the same conditions of 60 N 5 Hz, respectively (Figures S4 and S5 in the Supplementary Materials). This enhancement may be due to the larger stress subjected onto the BaTiO_3_ NPs under the reduced thickness upon stretching. The output voltage will not be affected by reducing the number of BaTiO_3_ NPs since the device can be considered as a matrix of parallelly connected units. When it is stretched, the number of units connected in parallel is reduced since the force is exerted in a fixed 1 cm^2^ area. In piezoelectric material, the current is induced from the external circuit by the potential generated from the internal polarization. Therefore, considering the area of the sample under compression force, the current density will not decrease with the decrease in the number of BaTiO_3_ NPs. A similar increase in power output has been earlier found in a stretchable hydrogel-based triboelectric nanogenerator [43]. Furthermore, the output voltage of the device under compressive force ranging from 30 to 60 N was measured at an un-strained state (Figure 5a,b). The output voltage linearly increases with the input force with a sensitivity of 59.8 mV/N, which is similar to some reported BaTiO_3_ and PZT self-powered sensors for body motion sensing [3,44]. After stretching, the sensitivity slightly increases (Figure 5b, Figures S6 and S7 in the Supplementary Materials), which may be due to the less damping from the elastomers to transfer more force onto the BaTiO_3_ nanoparticles. The linear increase in output voltage versus input force shows a good sensing behavior, which shows the potential for this stretchable piezoelectric nanogenerator to act as a wearable sensor for body motion sensing and gait sensing. To demonstrate the gait sensing potential, the piezoelectric nanogenerator was mounted on the bottom of the human heel to sense the foot stepping motion and frequency as a self-powered physiological sensor (Figure S8 in the Supplementary Materials).

## 4. Conclusions

In summary, a stretchable piezoelectric nanogenerator was developed with BaTiO_3_ NPs and photocurable elastomers EAA and AUD, that remain to be applied in printable piezoelectric energy applications. Both piezoelectric materials and electrodes are 3D printed, which makes them the first 3D printed stretchable piezoelectric nanogenerator. Excellent stretchability has been achieved by forming the composite by combining BaTiO_3_ NPs and the photocurable elastomer. The maximum strain for the printed BaTiO_3_ NP/EAA/AUD sample is 434%. This device generates an output voltage of 0.29 V, the current density is 0.20 μA/cm^2^ and the calculated power density is 57 nW/cm^2^. Furthermore, this stretchable piezoelectric nanogenerator has a sensitivity of 59.8 mV/N, and it was demonstrated to real-time monitor the foot stepping signal, which indicates its potential to be used as a self-powered body motion or gait sensor for stretchable or wearable electronic systems. 

## Supplementary Materials

The following are available online at https://www.mdpi.com/1424-8220/20/23/6748/s1. Figure S1: Schematic of the air bubble removal by the sliding process in DLP, Figure S2: SEM image of the cross-section of the device, Figure S3: Cyclic tensile testing of the BaTiO3 NP/EAA/AUD composite (three cycles), Figure S4: Output voltage under 60 N force and 5 Hz frequency of stretched sample at 100 % strain, Figure S5: Output voltage under 60 N force and 5 Hz frequency of stretched sample at 100 % strain, Figure S6: Output voltage under 30 N to 60 N forces and 5 Hz frequency of stretched sample at 50 % strain, Figure S7: Output voltage under 30 N to 60 N forces and 5 Hz frequency of stretched sample at 100 % strain, Figure S8: PENG device used for self-powered physiological monitoring of foot stepping.

## References

[1] Armaroli N., Balzani V. (2007). The future of energy supply: Challenges and opportunities. Angew. Chem. Int. Ed..

[2] Jeong C.K., Lee J., Han S., Ryu J., Hwang G.T., Park D.Y., Park J.H., Lee S.S., Byun M., Ko S.H. (2015). A hyper-stretchable elastic-composite energy harvester. Adv. Mater..

[3] Chen X., Parida K., Wang J., Xiong J., Lin M.-F., Shao J., Lee P.S. (2017). A Stretchable and Transparent Nanocomposite Nanogenerator for Self-Powered Physiological Monitoring. ACS Appl. Mater. Interfaces.

[4] Zhou X., Parida K., Halevi O., Liu Y., Xiong J., Magdassi S., Lee P.S. (2020). All 3D-printed stretchable piezoelectric nanogenerator with non-protruding kirigami structure. Nano Energy.

[5] Fan F.R., Tang W., Wang Z.L. (2016). Flexible Nanogenerators for Energy Harvesting and Self-Powered Electronics. Adv. Mater..

[6] Parida K., Thangavel G., Cai G., Zhou X., Park S., Xiong J., Lee P.S. (2019). Extremely stretchable and self-healing conductor based on thermoplastic elastomer for all-three-dimensional printed triboelectric nanogenerator. Nat. Commun..

[7] Lin M.-F., Thakur V.K., Tan E.J., Lee P.S. (2011). Dopant induced hollow BaTiO3 nanostructures for application in high performance capacitors. J. Mater. Chem..

[8] Zhou Z., Bowland C.C., Patterson B.A., Malakooti M.H., Sodano H.A. (2016). Conformal BaTiO_3_ Films with High Piezoelectric Coupling through an Optimized Hydrothermal Synthesis. ACS Appl. Mater. Interfaces.

[9] Schult M., Buckow E., Seitz H. (2016). Experimental studies on 3D printing of barium titanate ceramics for medical applications. Curr. Dir. Biomed. Eng..

[10] Jeong C.K., Kim I., Park K.I., Oh M.H., Paik H., Hwang G.T., No K., Nam Y.S., Lee K.J. (2013). Virus-directed design of a flexible BaTiO3 nanogenerator. ACS Nano.

[11] Kim H., Torres F., Islam M.T., Islam M.D., Chavez L.A., Garcia Rosales C.A., Wilburn B.R., Stewart C.M., Noveron J.C., Tseng T.L.B. (2017). Increased piezoelectric response in functional nanocomposites through multiwall carbon nanotube interface and fused-deposition modeling three-dimensional printing. MRS Commun..

[12] Siddiqui S., Kim D.-I., Duy L.T., Nguyen M.T., Muhammad S., Yoon W.S., Lee N.E. (2015). High-performance flexible lead-free nanocomposite piezoelectric nanogenerator for biomechanical energy harvesting and storage. Nano Energy.

[13] Patel D.K., Sakhaei A.H., Layani M., Zhang B., Ge Q., Magdassi S. (2017). Highly Stretchable and UV Curable Elastomers for Digital Light Processing Based 3D Printing. Adv. Mater..

[14] Frey M.H., Payne D.A. (1996). Grain-size effect on structure and phase transformations for barium titanate. Phys. Rev. B.

[15] Chen Z., Song X., Lei L., Chen X., Fei C., Chiu C.T., Qian X., Ma T., Yang Y., Shung K. (2016). 3D printing of piezoelectric element for energy focusing and ultrasonic sensing. Nano Energy.

[16] Ehterami A., Kazemi M., Nazari B., Saraeian P., Azami M. (2018). Fabrication and characterization of highly porous barium titanate based sca ff old coated by Gel / HA nanocomposite with high piezoelectric coe ffi cient for bone tissue engineering applications. J. Mech. Behav. Biomed. Mater..

[17] Gaytan S.M., Cadena M.A., Karim H., Delfin D., Lin Y., Espalin D., MacDonald E., Wicker R.B. (2015). Fabrication of barium titanate by binder jetting additive manufacturing technology. Ceram. Int..

[18] Martirena H.T., Burfoot J.C. (1974). Grain-size effects on properties of some ferroelectric ceramics. J. Phys. C Solid State Phys..

[19] Mangalam R.V.K., Ray N., Waghmare U.V., Sundaresan A., Rao C.N.R. (2009). Multiferroic properties of nanocrystalline BaTiO_3_. Solid State Commun..

[20] Wang J., Cai G., Li S., Gao D., Xiong J., Lee P.S. (2018). Printable Superelastic Conductors with Extreme Stretchability and Robust Cycling Endurance Enabled by Liquid-Metal Particles. Adv. Mater..

[21] Qi Y., Kim J., Nguyen T.D., Lisko B., Purohit P.K., McAlpine M.C. (2011). Enhanced piezoelectricity and stretchability in energy harvesting devices fabricated from buckled PZT ribbons. Nano Lett..

[22] Lionel Y.L., Fedder G.K. Elastic ribbon-like piezoelectric energy harvester for wearable devices with stretchable surfaces. Proceedings of the 2016 38th Annual International Conference of the IEEE Engineering in Medicine and Biology Society (EMBC).

[23] Yun D., Yun K.-S. (2013). Woven piezoelectric structure for stretchable energy harvester. Electron. Lett..

[24] Lee J.H., Lee K.Y., Gupta M.K., Kim T.Y., Lee D.Y., Oh J., Ryu C., Yoo W.J., Kang C.Y., Yoon S.J. (2014). Highly stretchable piezoelectric-pyroelectric hybrid nanogenerator. Adv. Mater..

[25] Park S.H., Lee H.B., Yeon S.M., Park J., Lee N.K. (2016). Flexible and Stretchable Piezoelectric Sensor with Thickness-Tunable Configuration of Electrospun Nanofiber Mat and Elastomeric Substrates. ACS Appl. Mater. Interfaces.

[26] Duan Y., Ding Y., Bian J., Xu Z., Yin Z., Huang Y. (2017). Ultra-stretchable piezoelectric nanogenerators via large-scale aligned fractal inspired micro/nanofibers. Polymers.

[27] Qian S., Qin L., He J., Niu X., Qian J., Mu J., Geng W., Hou X., Chou X. (2019). A stretchable piezoelectric elastic composite. Mater. Lett..

[28] Chou X., Zhu J., Qian S., Niu X., Qian J., Hou X., Mu J., Geng W., Cho J., He J. (2018). All-in-one filler-elastomer-based high-performance stretchable piezoelectric nanogenerator for kinetic energy harvesting and self-powered motion monitoring. Nano Energy.

[29] Bian J., Wang N., Ma J., Jie Y., Zou J., Cao X. (2018). Stretchable 3D polymer for simultaneously mechanical energy harvesting and biomimetic force sensing. Nano Energy.

[30] Siddiqui S., Lee H.B., Kim D.-I., Duy L.T., Hanif A., Lee N.E. (2018). An Omnidirectionally Stretchable Piezoelectric Nanogenerator Based on Hybrid Nanofibers and Carbon Electrodes for Multimodal Straining and Human Kinematics Energy Harvesting. Adv. Energy Mater..

[31] Ando B., Baglio S., Bulsara A.R., Marletta V., Ferrari V., Ferrari M. (2015). A low-cost snap-through-buckling inkjet-printed device for vibrational energy harvesting. IEEE Sens. J..

[32] Rezaeisaray M., El Gowini M., Sameoto D., Raboud D., Moussa W. (2015). Low frequency piezoelectric energy harvesting at multi vibration mode shapes. Sens. Actuators A Phys..

[33] Wang X., Kim K., Wang Y., Stadermann M., Noy A., Hamza A.V., Yang J., Sirbuly D.J. (2010). Matrix-Assisted Energy Conversion in Nanostructured Piezoelectric Arrays. Nano Lett..

[34] Saravanakumar B., Kim S. (2014). Growth of 2D ZnO Nanowall for Energy Harvesting Application. J. Phys. Chem. C.

[35] Rahman A., Lee B., Phan D. (2013). Fabrication and characterization of highly efficient flexible energy harvesters using PVDF—graphene nanocomposites. Smart Mater. Struct..

[36] Sim H.J., Choi C., Lee C.J., Kim Y.T., Kim S.J. (2015). Flexible Two-ply Piezoelectric Yarn Energy Harvester. Curr. Nanosci..

[37] Briscoe J., Dunn S. (2014). Piezoelectric nanogenerators—A review of nanostructured piezoelectric energy harvesters. Nano Energy.

[38] Wang H., Mercier P.P. (2017). Near-Zero-Power Temperature Sensing via Tunneling Currents Through Complementary Metal- Oxide-Semiconductor Transistors. Sci. Rep..

[39] Tekeste T., Saleh H., Member S., Mohammad B., Member S. (2018). A Nanowatt Real-Time Cardiac Autonomic Neuropathy Detector. IEEE Trans. Biomed. Circuits Syst..

[40] Harpe P., Member S., Gao H., Van Dommele R., Cantatore E., Member S., Van Roermund A.H.M., Member S. (2016). A 0.20 mm^2^ 3 nW Signal Acquisition IC for Miniature Sensor Nodes in 65 nm CMOS. IEEE J. Solid-State Circuits.

[41] Pinrod V., Pancoast L., Davaji B., Lee S., Ying R., Molnar A., Lal A. Zero-power sensors with near-zero-power wakeup switches for reliable sensor platforms. Proceedings of the 2017 IEEE 30th International Conference on Micro Electro Mechanical Systems.

[42] Nazari M.H., Mujeeb-v-rahman M., Scherer A. An Implantable Continuous Glucose Monitoring Microsystem in O.18f.1m CMOS. Proceedings of the 2014 IEEE Symposium VLSI Circuits Digest Technical Reports.

[43] Parida K., Kumar V., Jiangxin W., Bhavanasi V., Bendi R., Lee P.S. (2017). Highly Transparent, Stretchable, and Self-Healing Ionic-Skin Triboelectric Nanogenerators for Energy Harvesting and Touch Applications. Adv. Mater..

[44] Zhu M., Shi Q., He T., Yi Z., Yang B., Chen T., Lee C. (2019). Self-Powered and Self-Functional Cotton Sock Using Piezoelectric and Triboelectric Hybrid Mechanism for Healthcare and Sports Monitoring. ACS Nano.

