# Supplementary material for: All 3D Printed Stretchable Piezoelectric Nanogenerator for Self-Powered Sensor Application
Source: Sensors (Basel). 2020 Nov 26;20(23):6748. doi: 10.3390/s20236748 (PMC7728330; doi:10.3390/s20236748)
Supplement: Supplementary file 1 [file sensors-20-06748-s001.pdf]

Supporting Information

for

# All 3D-printed stretchable piezoelectric nanogenerator for self-powered sensor application

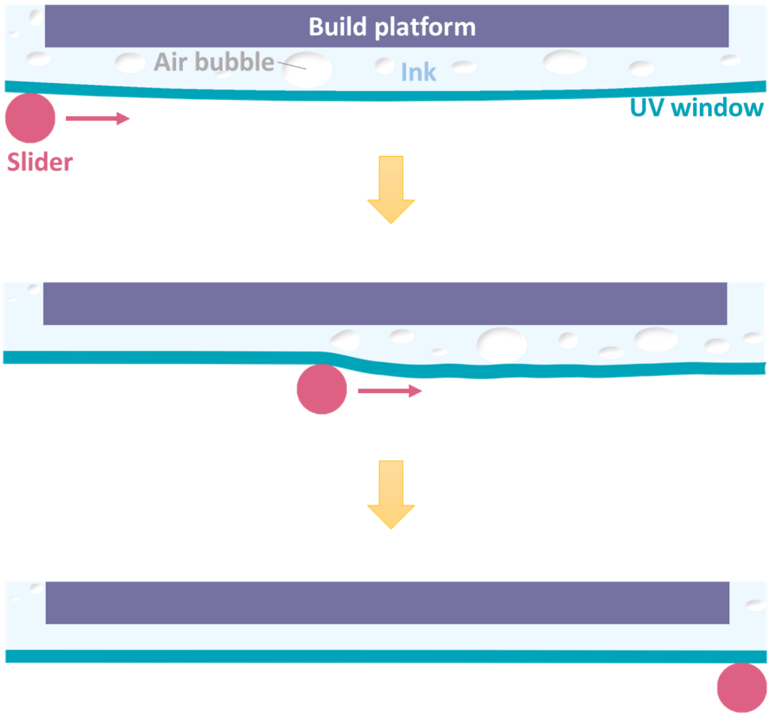


Figure S1. Schematic of the air bubble removal by the sliding process in DLP.

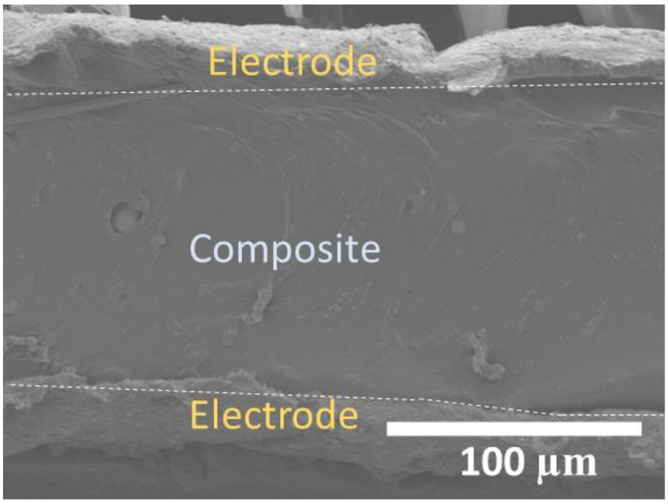


Figure S2. SEM image of the cross-section of the device.

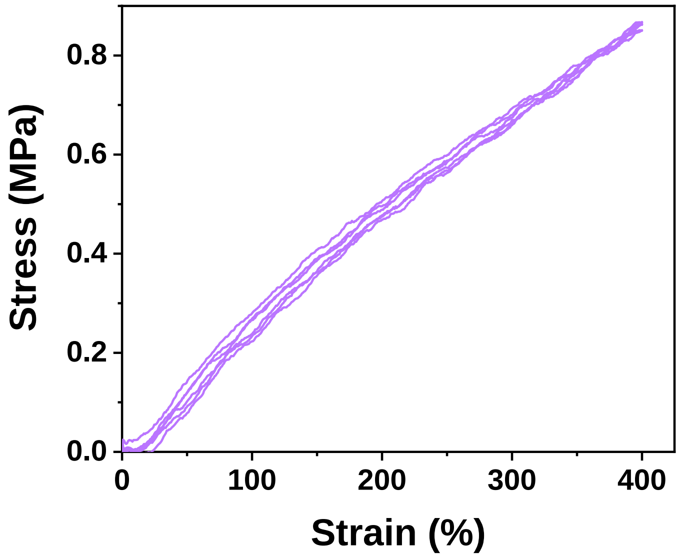


Figure S3. Cyclic tensile testing of the $BaTiO_3$ NP/EAA/AUD composite (three cycles).

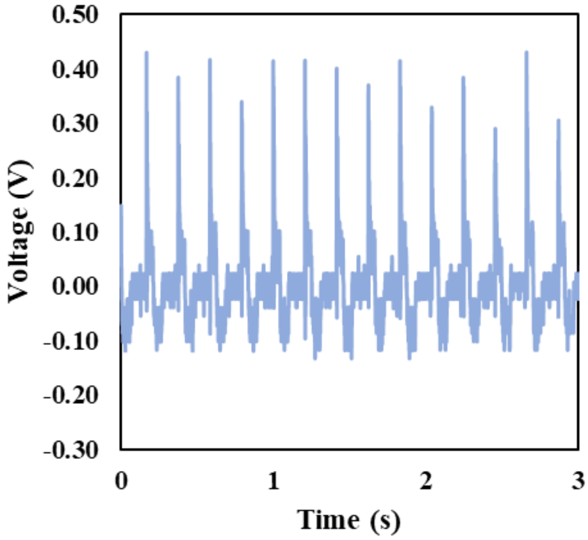


Figure S4. Output voltage under 60 N force and 5 Hz frequency of stretched sample at 100 % strain.

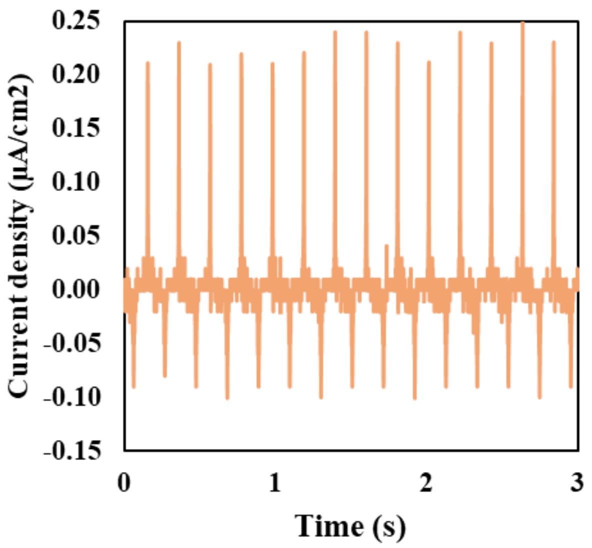


Figure S5. Output voltage under 60 N force and 5 Hz frequency of stretched sample at 100 % strain.

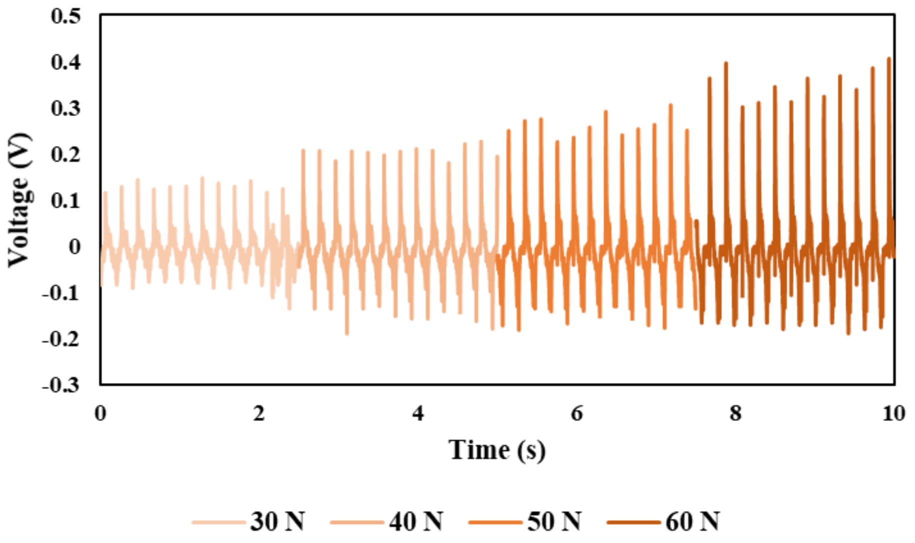


Figure S6. Output voltage under 30 N to 60 N forces and 5 Hz frequency of stretched sample at 50 % strain.

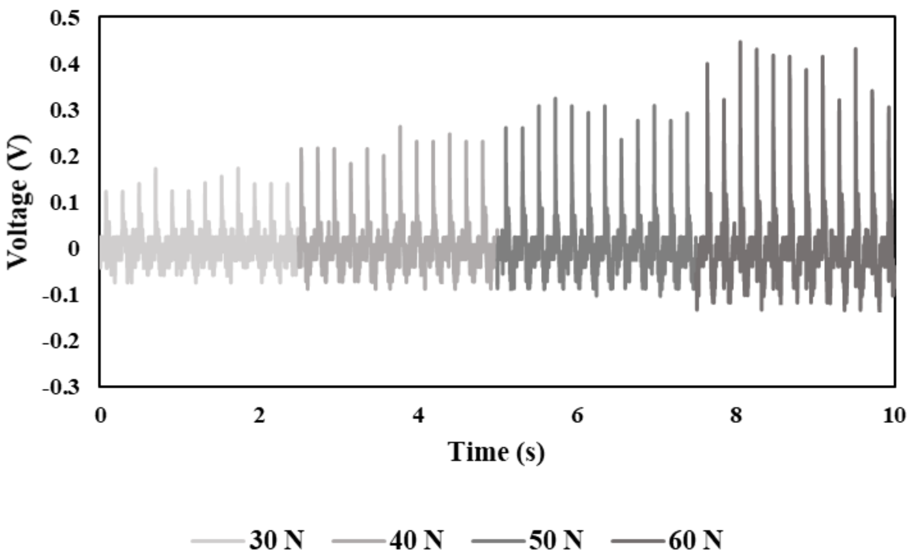


Figure S7. Output voltage under 30 N to 60 N forces and 5 Hz frequency of stretched sample at 100 % strain.

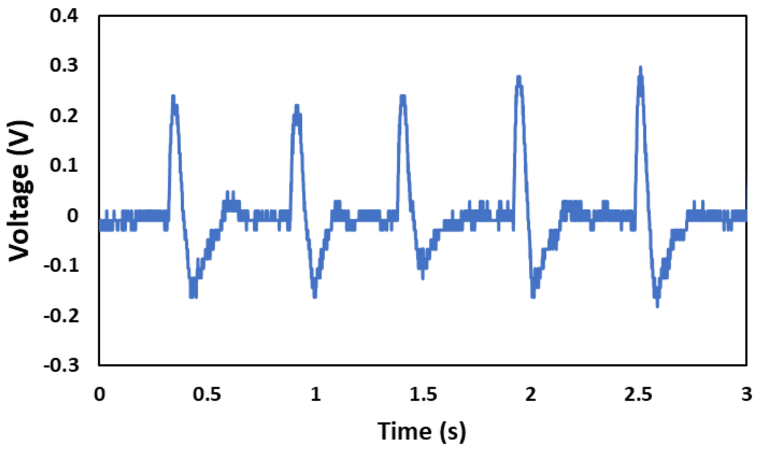


Figure S8. PENG device used for self-powered physiological monitoring of foot stepping.
